# Green Synthesis of Silver Nanoparticles as an Effective Antibiofouling Material for Polyvinylidene Fluoride (PVDF) Ultrafiltration Membrane

**DOI:** 10.3390/polym13213683

**Published:** 2021-10-26

**Authors:** Nour Alnairat, Muna Abu Dalo, Rund Abu-Zurayk, Saida Abu Mallouh, Fadwa Odeh, Abeer Al Bawab

**Affiliations:** 1 Chemistry Department, The University of Jordan, Amman 11942, Jordan; Nouralnairat1991@gmail.com (N.A.); f.odeh@ju.edu.jo (F.O.); 2 Chemistry Department, Jordan University of Science and Technology, Irbid 22110, Jordan; 3 Hamdi Mango Center for Scientific Research, The University of Jordan, Amman 11942, Jordan; r.abuzurayk@ju.edu.jo (R.A.-Z.); s.mallouh@ju.edu.jo (S.A.M.); 4 The Nanotechnology Center, The University of Jordan, Amman 11942, Jordan

**Keywords:** silver nanoparticles, green synthesis, PVDF, ultrafiltration, antibiofouling

## Abstract

Silver nanoparticles (AgNPs) were successfully synthesized using the aqueous extract of the *Paronychia argentea* Lam (*P. argentea*) wild plant. The results showed that the conversion of Ag^+^ to Ag^0^ nanoparticles ratio reached 96.5% as determined by Inductively Coupled Plasma Atomic Emission Spectroscopy (ICP-AES), with a negative zeta potential (ζ) of −21.3 ± 7.68 mV of AgNPs expected to improve the stability of synthesized AgNPs. AgNP antibacterial activity has been examined against *Streptococcus aureus* (*S. aureus*) and *Escherichia coli* (*E. coli*) bacteria. The minimum inhibition concentration (MIC) was 4.9 µL/mL for both *E. coli* and *S. aureus* bacteria, while the minimum bactericidal concentrations (MBC) were 19.9 µL/mL and 4.9 µL/mL for *S. aureus* and *E. coli*, respectively. The synthesized AgNPs were incorporated in ultrafiltration polyvinylidene Fluoride (PVDF) membranes and showed remarkable antibiofouling behavior against both bacterial strains. The membranes were characterized using Fourier transform infrared spectroscopy (FTIR), scanning electron microscope (SEM), and X-ray diffraction (XRD). The contact angle and porosity of the membrane were also determined. The efficiency of the membranes regarding rejection rate was assessed using bovine serum albumin (BSA). It was found in the flux experiments that membranes BSA rejection was 99.4% and 98.7% with and without AgNPs, respectively.

## 1. Introduction

The population boom and the process of urbanization in the recent hundreds of years had caused considerable shortage of fresh water [1,2]. Jordan is ranked as one of the most water-scarce countries in the world, with most of its regions being arid and semiarid, with large temporal and spatial variations in rainfall proportions [3]. Owing to the continuous rise in the population of Jordan, whether due to natural growth or continuous refugees’ waves due to the volatile conditions in the region, water scarcity has become a serious issue [4]. The regeneration of fresh water from saline water supplies will probably be a long-term solution for this issue. Membrane technology has increased in popularity as a result of the advantages it provides in water treatment [5]. Compared to other chemical and thermal methods, membrane technology follows sustainability standards in terms of environmental effects, ease of use, land use, flexibility, and adaptability [6]. Ultrafiltration (UF) membranes are widely utilized for purification and separation of wastewater generated from pharmaceutical, biological, and food industries. UF membranes can remove organic particles, bacteria, and proteins from a liquid stream, which are considered as very significant pollutants in water. Among the various polymeric-based membranes available, polyvinylidene fluoride (PVDF) membrane has gained a lot of interest due to its outstanding chemical stability, corrosion resistance, and thermal stability [7,8,9,10]. However, PVDF fouling throughout separation processes is considered a major problem due to its hydrophobic nature [11,12]. The fouling of membranes can be controlled by a selection of suitable modifications [13], such as physical blending [14,15,16,17], plasma treatment [18,19], artificial graft modification [20,21], and surface coating [22,23,24]. Plasma treatment and chemical grafting methods generate durable membranes, but they cause variations in the main chain of polymer and lower the water treatment efficiency. On the other hand, the toughness of the coating layer is often a concern in surface coating. Physical mixing is appealing since phase inversion is handy to prepare, and blend modification is utilized for large-scale industrial production [13]. Nanoparticles (NPs) have been considered a useful way to enhance the hydrophilicity, antibacterial, and antifouling membrane properties [25,26,27]. AgNPs have been shown to be very active antibacterial agents [28] and antifungal agents [29,30,31] because of their high cytotoxic effect against a variety of microorganisms, especially in comparison with other metal nanoparticles [32] and antioxidants [33]. Recently, aqueous honey solutions with concentrations of 2%, 10%, and 20% were used to synthesize AgNPs to study their activity against fungi; the results show the activity of AgNPs against fungal is directly related to the concentration of honey utilized in their synthesis [31]. Ahsani and coworkers prepared modified PVDF and PVDF/Ag-SiO_2_ membranes, where silica nanoparticles were used as a support for AgNPs to insure good separation and homogeneity of the nanocomposite [32]. The PVDF/Ag-SiO_2_ showed enhanced antiadhesion and antibacterial properties due to the presence of AgNPs. In a recent study by Abduraimova and coworkers, the AgNPs were linked to the surface of mesoporous silica nanoparticles (MSN) loaded with cetyltrimethylammonium bromide (CTAB). Due to the antibacterial properties of both CTAB and AgNPs, the nanocomposite showed unprecedented antibacterial effect when recalculated to Ag weight in the composite [34].

Chemical, physical, and biological approaches have been used to synthesize NPs [35]. Primary sources for the biosynthesis of NPs include microorganisms (fungi, yeast, bacteria, actinomycete, and viruses) and synthesis mediated by plants’ extract [36]. Synthesized NPs with plant extract seem to be more beneficial than intracellular microorganism synthesis due to the complications of the latter method such as isolation, culture maintenance, and multiple purification steps. On the other hand, green techniques utilizing various plant parts, such as root [37], leaf [35,37,38], flower [38], peel [39], and fruit [40] have been developed [41]. In addition, some researchers used dry plasma reduction as a green method to prepare NPs [42]. Flavonoids, polyphenols, proteins, ascorbic acid, and terpenoids are the main compounds found in plant extracts that act as metal ion adsorbents, precursor salt-reducing agents, and capping agents, some of which have actual antimicrobial activities [41].

Modifications of PVDF membranes by the addition of green nanoparticles are getting more attention due to their effect in enhancing antimicrobial, antifouling, and hydrophilic properties of those membranes. PVDF membranes embedded with AgNPs using apple extract and gallic acid from pomegranate peels as reducing agents showed an obvious bacterial growth inhibition on several types of both Gram-positive and Gram-negative bacteria [43,44]. Furthermore, the hydrophilicity of PVDF surface was also enhanced when membranes were loaded with synthesized AgNPs using ginger extracts [43], which led to a lower contact angel by around 8 degrees [45]. In parallel, the modified PVDF membranes were evaluated for their antifouling properties using bovine serum albumin (BSA) filtration test which proved that the rejection rate of BSA solution exceeded 68% [43,46].

In this study, AgNPs were synthesized using the extract of *P. argentea* (a wild plant available in Jordan) as reducing and stabilizing agent. The synthesized AgNPs were incorporated into membrane casting solutions, followed by PVDF membranes fabrication by phase inversion method. The PVDF ultrafiltration nanocomposite membranes were evaluated for their antimicrobial, flux, and rejection properties. To the best of our knowledge, no previous studies have reported the synthesis of AgNPs using *P. argentea* extract and their use in fabrication of PVDF nanocomposite membranes as an efficient antibiofouling material.

## 2. Materials and Methods

### 2.1. Materials

*P. argentea* was collected in the spring (2019) from Ajloun Forest Reserve (north of Jordan) and from Khalda (western Amman). Silver nitrate (AgNO3) (99.0%, Bio Basic Inc., ACS, Amherst, New York, USA), polyvinylidene fluoride (PVDF) average molecular weight 534,000 (Arkema, Colombes, France), N, N-dimethylacetamide (DMAA) HPLC/Spectro (Tedia, Fairfield, OH, USA). Polyvinylpyrrolidone (PVP) average molecular weight 36,000 (Sigma-Aldrich, Beijing, China) bovine serum albumin (BSA) Lypohilised pH~7 (Biowest, Nuaille, France), Whatman filter paper No.1, preclean syringe filters, and deionized water (DW) have been used in this study.

For antibacterial tests, nutrient agar, meat extract, peptone, casein peptone, and soybean peptone were purchased from Liofilchem, Roseto, Italy; nutrient broth (Oxoid, Mumbai, India), sodium chloride (extra pure, Lobachemie, Mumbai, India), D-glucose (JHD, Guangdong, China), lecithin, and di-kaliumhydrogenphosphate from Riedel de-Haen AG Seelze–Hannover, Germany; tryptone soya agar (casein soya bean digest medium) (Oxoid, Hampshire, UK); Tween 80 (Polysorbate-80) (ICI, London, UK); yeast extract agar (Mast Group, Merseyside, UK); agar (Merck, Branchburg, NJ, USA); and tryptone powder (Bio Basic, Markham, ON, Canada). In addition, a Gram-negative *Escherichia coli* (*E. coli*) bacteria and Gram-positive *Staphylococcus aureus* (*S. aureus*) bacteria used were ATCC no. 8739 and 25913, respectively.

### 2.2. Methods

#### 2.2.1. Synthesis of AgNPs

##### Preparation of Aqueous *P. argentea* Extract

*P. argentea* (aerial parts) were dried in oven at 50–70 °C, ground as a powder, and stored for later use in a desiccator. A total of 100.0 mL of DW was added to 5.0 g of dried *P. argentea* powder. The aqueous extract was boiled for 20 min, followed by filtration with Whatman filter paper No.1; successive filtrations were done with preclean syringe filter (size 0.45 μm) [47].

##### Preparation of AgNPs

In the dark at room temperature, the aqueous extract of *P. argentea* was added dropwise using Pasteur pipette into a 50.0 mL of 3.0 mM solution of AgNO_3_ at a rate of 2.0 mL/h while stirring using a magnetic stirrer. Various volumes of aqueous *P. argentea* extract (6.0, 8.0, 10.0, and 12.0 mL) and different magnetic stirrer rotation (350, 700, 1000, and 1500 rpm) were tested for AgNPs synthesis optimization. At room temperature, the reaction mixture was continuously stirred until all plant extract was added, with an extra hour of stirring after the final addition. The aqueous solution color changed from pale yellow to dark brown, indicating that the Ag^+^ ions had been reduced to Ag^0^ (AgNPs). The prepared AgNPs suspension was stored at 4.0 °C.

#### 2.2.2. Polyvinylidene Fluoride (PVDF) Casting Process

PVDF nanocomposite (PVDF/NC) membrane (with AgNPs) was prepared by blending the synthesized AgNPs with 76.34 g DMAA; then 22.0 g PVDF and 1.0 g PVP were added gradually with stirring at 100.0 rpm for 6.0 h at 70–80 °C. The solution was degassed for 12 h to produce gel utilizing a rod. Film casting was formed on a glass plate; then the film was immersed in a bath of DW at a temperature of 25 ± 1 °C until membrane was formed by phase inversion. The membranes were then washed to eliminate any remaining solvent and stored in a bath of DW until testing and characterization [48].

Without the addition of AgNPs, the PVDF standard membrane PVDF/PURE was prepared using the same procedure as the PVDF/NC membrane.

#### 2.2.3. Characterization

UV-Vis spectrophotometry (Cary 100, Santa Clara, California, USA), ranging 200–800 nm, was used to examine the presence of AgNPs at λ = 420 nm and the concentration of BSA utilized in the PVDF rejection test experiment at λ= 280 nm.

AgNPs conversion ratio was determined using inductively coupled plasma/atomic emission spectroscopy (ICP/AES) (GBC E1475, Hampshire, MA, USA).

Dynamic light scattering (DLS) (Zetatrac, Westborough, MA, USA) was used to measure the average hydrodynamic diameter, size distribution, zeta potential, and polydispersity index (PDI) for AgNP suspension.

Transmission electron microscope (TEM) (JEOI JEM2100, North of Boston, MA, USA) at resolution point of 0.25 nm, Lattice 0.14 nm, STEM 1.0 nm, and using Gatan 2 k × 2 k digital camera was used to determine the particle size of AgNPs and their distribution in the polymeric matrix of the fabricated membranes.

Attenuated total reflectance–Fourier transfer infrared spectroscopy (ATR–FTIR) spectrophotometer (Thermo Nicolet NEXUS 670, Watertown, MA, USA) was used to detect AgNP interactions with plant extract biomolecules as well as AgNPs effects on polymer membranes at range between 4000–400 cm^−1^, resolution of about 4.0 cm^−1^, and the scan numbers of 32.

Freeze dryer (CHRIST, ALPHA 2-4 LD PLUS, New York, NY, USA) was used to convert AgNPs solution to AgNPs powder.

The crystalline structure of AgNPs and membranes and the effect of AgNPs on the crystalline structure of membranes were studied using X-ray diffraction (XRD). The XRD patterns of AgNPs and membranes were looked at using a 7000 Shimadzu 2 kW model X-ray spectrophotometer instrument (Japan) with a nickel filtered copper radiation (CuKa) with λ = 1.5456 Å, 2 θ ranging (2°–90°) with a step size of 0.02°.

Finally, the pore size and morphology of membranes surface was tested by Scanning electronic microscope (SEM) (FEI Company; Inspect F50 High Vacuum 6 × 10^−4^ Pa, Eindhoven, NB, USA).

Water contact angle measurement was used to determine the hydrophilicity of the membrane surface by contact angle meter (Attension, Biolin Scientific, Manchester, UK). The sessile drop approach was used, and DW was utilized as the probe liquid to examine the contact angles between DW and the membrane’s surface; after the DW was dropped on the membrane’s airside surface for 10 s, the contact angle was determined.

At a 1.0 bar transmembrane pressure and room temperature, pure water flux and BSA rejection were determined using the dead-end cell system (Millipore Sigma, Amicon, Santa Clara, USA) for membranes with an area of 28.26 cm^2^ at 350 rpm stirring and using 0.5 g/L BSA. Each membrane was first prepressed for 40.0 min. with DW at 1.0 bar. The flux rate was determined utilizing DW; after that, the aqueous solution of BSA was permeated for 30.0 min. The BSA concentration permeation and feed solution were determined using a UV-Vis spectrophotometry at a wavelength of 278.0 nm.

Permeation fluxes (J_w_) and BSA rejection (R) were calculated using the following equations:

(1) $$J_{w}=\frac{V}{A.∆t}$$

(2) $$R\;=(1-\frac{\mathrm{CP}}{\mathrm{CF}})\;\times \;100\%$$

where:

V (L): Water permeate volume.

A (m^2^): Membrane effective area.

∆t (h): Time of permeability.

CP: BSA permeation concentration.

CF: Feed solution.

Knowing that CF was kept at 0.5 g/L in a permeation solution, CP was checked after 30.0 min [48].

Two tests were performed to test AgNP release from the membranes: first, PVFD/NC membranes were soaked in DW for two months; after that, the water was tested for presence of AgNPs using ICP/AES. In the second test, two samples of PVDF/NC membrane were used in the permeability test by DW using dead-end cell at 1.0 bar. The AgNPs concentration of the filtrate was tested for presence of AgNPs after 40.0 min by ICP/AES [49].

The gravimetric method was utilized to determine the porosity of the membranes. Three samples of two types of membranes PVDF/PURE and PVDF/NC were saturated with DW, then weighed after removing excess water from the surfaces using filter paper (m_1_). The membranes were then dried for 48 h at room temperature, and their weights were determined (m_2_). The membrane porosity (ε) % was calculated using the following Equation [50,51]:

(3) $$\varepsilon =\frac{\left(m_{1}-m_{2}\right)/pH_{2}O}{\frac{m_{1}-m_{2}}{pH_{2}O}+m_{2}/\mathrm{pP}}$$

where:

ε: Membrane porosity.

m_1_: The weight of wet sample.

m_2_: The weight of dry sample.

pH_2_O: Deionized water density (1.0 g/cm^3^).

pP: Polymer (PVDF) density (1.74 g/cm^3^).

### 2.3. Antibacterial Activity

#### 2.3.1. Antibacterial Activity for AgNPs

The antibacterial activity for AgNPs was tested using the microdilution assay. It was utilized mainly in the determination of minimum inhibition concentration (MIC), which is the least concentration of substance that prevents visible growth of bacteria, and minimum bactericidal concentration (MBC), which is the minimum concentration that can prevent the growth of bacteria or kill bacteria. Microdilution assay was utilized to determine the antibacterial activity of aqueous extract of *P. argentea*, AgNO_3_, and AgNPs against *E. coli* and *S. aureus*. Each concentration of bacteria was maintained to approximately 6 × 10^6^ bacteria/mL for the test of MIC and MBC. After that, 100.0 μL of every test sample (*P. argentea* aqueous extract, AgNO_3_, AgNPs) were diluted in serials, in a 96-well plate with dilutions from 1:1 to 1:10. The nutrient broth was utilized as a negative control. Positive or negative control were utilized to ensure sufficient bacterial growth and media sterility, respectively. The plates were then incubated at 37.0 °C for 24 h in a plate shaker incubation. After incubation, the MIC for each sample was visually evaluated based on turbidity so that the MIC was determined to be the concentration in the last clear well. Regarding MBC, a loop was taken from the first well after the MIC well to be cultured on the plates of nutrient agar at 37.0 °C for 24 h, then the results of MBC were obtained.

#### 2.3.2. Membrane Antibacterial Activity

The antibacterial activity of the membranes was tested using the standard “ISO 22196:2007 Plastics—Measurement of antibacterial activity on plastics surfaces”.

##### Preparation of Culture Media and Solutions

Nutrient broth (13.0 g) in 1.0 L DW was used to prepare nutrient broth. First, 28.0 g of nutrient agar in 1.0 L of DW was used to prepare nutrient agar. Then, 1.0 g of glucose, 5.0 g of tryptone, 2.5 g of yeast extract, and 15.0 g of agar powder in 1.0 L of DW were used to prepare plate count agar. A total of 3.0 g of soybean peptone, 17.0 g of casein peptone, 2.5 g of disodium hydrogen phosphate, 5.0 g of sodium chloride, 1.0 g of lecithin, 2.5 g of glucose, and 7.0 g of nonionic surfactant in 1.0 L DW were used to prepare soybean casein digest broth with lecithin and polyoxyethylene sorbitan monooleate (SCDLP broth). The pH was adjusted to 6.8–7.2. We used 34.0 g of potassium dihydrogen phosphate in 1.0 L DW to prepare the solution of phosphate buffer at pH of 6.8–7.2. Then, 8.5 g of sodium chloride in 1.0 L of DW was used to prepare phosphate-buffered physiological saline. The solution of phosphate buffer was diluted with the physiological saline to an 800-fold volume. Autoclaving was used to sterilize all the media. All media was dissolved in <1 μS/cm conductivity deionized water.

##### Preparation of Membrane Samples

Three samples of PVDF/NC membranes with dimensions of (2.2 × 2.2) cm^2^ had been utilized in parallel to six samples of control membrane (PVDF/PURE) with the same dimensions. Using 10.0 mL of SCDLP buffer, half of the untreated pieces (PVDF/PURE) were directly washed. After that, SCDLP was serially diluted to 10-fold in phosphate-buffer. A 100 µL of the previous dilution was taken and cultured on plate count agar for 24.0 h at 37.0 °C. The surviving pieces were washed in SCDLP buffer the next day, diluted, cultured, and incubated in the same manner as the untreated pieces. Triplicates of each plating steps were used. The number of colonies from each dilution was counted and reported after incubation. The number of viable bacteria recorded for each membrane sample was calculated using the Equation:

N = (100 × C × D × V)/A(4)

where:

N: the number of viable bacteria recovered per cm^2^ of membrane sample test.

C: the average plate counts for per the duplicate or triplicate plates.

D: the dilution factor of plates counted.

V: the volume (mL) of SCDLP added to the samples of membrane.

A: the surface area (mm^2^) of cover film.

If no colonies have been recovered in any of the agar plates for a dilution series, count the colonies as “V” (where V is the SCDLP volume (mL) addition to the membrane pieces).

When determining the average, describe “V” as the number of viable bacteria recovered when a dilution series had not recovered viable bacteria.

The average calculating number would be 10 in the case of V = 10 mL.

By the following Equation, the sterilization ratio was calculated:

(5) $$R=\frac{\left(A-B\right)}{A}\;\times 100$$

where:

R: the sterilization ratio.

A: the viable bacteria number recovered per cm^2^ of the untreated samples membrane (PVDF/PURE).

B: the viable bacteria number recovered per cm^2^ of the treated samples membrane (PVDF/NC) [7]

## 3. Results and Discussion

### 3.1. Optimization AgNPs Synthesis

The volume of aqueous *P. argentea* extract added and the percentage of conversion of Ag^+^ to AgNPs are summarized in Table 1. As shown in Table 1, the highest percentage of conversion was obtained when utilizing 8.0 mL of the aqueous *P. argentea* extract.

Thereafter, the aqueous *P. argentea* extract volume was fixed at 8.0 mL, while rpm was varied (350, 750, 1000, and 1500), as shown in Table 2.

Table 2 shows that the optimum conditions is 8.0 mL of aqueous *P. argentea* extract and 1000 rpm. Dropwise addition of aqueous *P. argentea* extract was maintained at 2 mL/h.

The reason of optimal condition at 1000 rpm is that the higher speeds produce smaller particles with lower average diameter in which more time is needed to reach the same conversation ratio (Ag+ to AgNPs (Ag^0^) [52].

Through the reduction process, AgNO_3_ solution color varied from clear to dark brown. This color change was considered to be an indication of the production of AgNPs [53]. Abu Dalo and coworkers synthesized AgNPs by dropwise addition method utilizing rosemary leaf extract (ROLEs) and olive leaf extracts (OLEs). The percentages of conversion of Ag^+^ to Ag^0^ (AgNPs) were found to be 53% for OLE–AgNPs and 48% for RLE–AgNPs based on atomic absorption spectrophotometric measurements [54].

#### 3.1.1. UV-Vis and DLS Measurements

**Figure 1:**
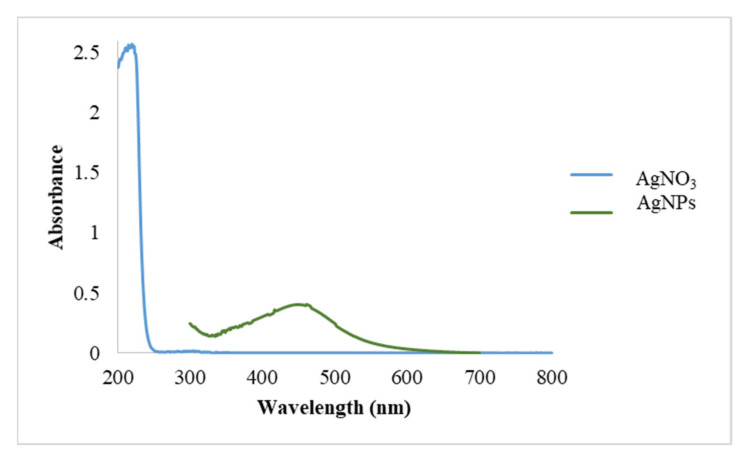
UV-Vis spectra of AgNO_3_ (aq) and AgNPs synthesized by aqueous extract of *P. argentea*.

The UV-Visible spectrum of AgNPs is shown in Figure 1 against AgNO_3_ solution (which was used as a blank). A λ_max_ was observed at 447 nm; this result agrees with values obtained for AgNPs prepared using plant extracts. For example, Atarod and colleagues prepared AgNPs from *Euphorbia heterophylla* leaves’ aqueous extract with λ_max_ at 440 nm [55]. In another study, AgNPs were produced by marine algae *Padina Sp* with λ_max_ values ranging between 420 nm and 445 nm [56].

**Figure 2:**
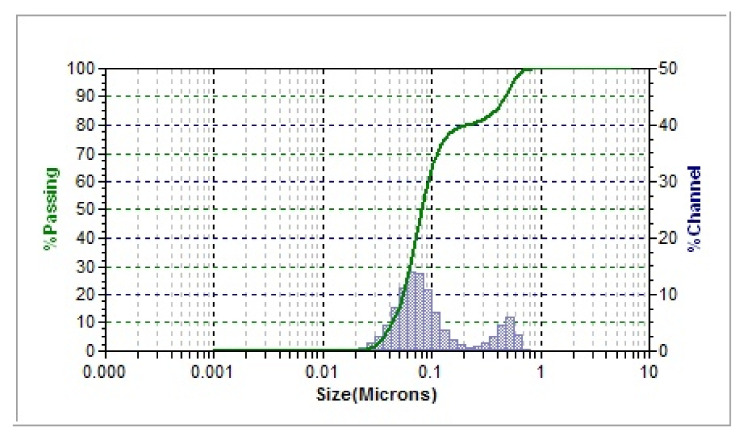
The distribution of intensity–hydrodynamic size for AgNPs synthesized by aqueous extract of *P. argentea* obtained from DLS.

Figure 2 shows the hydrodynamic size distribution for the samples prepared under optimized conditions. The average size was found to be 71.1 nm, and the polydispersity index (PDI) was 0.6920. The actual diameter of nanoparticles is predicted to be smaller than this value (71 nm) due to a solvent layer and the materials of surface coating attached to the surface of the particles as it moves under the Brownian motion influence [57]. As seen in Figure 2, there are two size distributions, the major population at ~70 nm, while the other one at ~0.5 μm, which are also present in the TEM image. The latter population can be attributed to several factors; one of them is the nature of technique used, weighing type, and sample treatment prior to DLS measurement. However, it is known that DLS measures hydrodynamic radius of whatever present in the sample due to lack of molecular identity sensitivity (compared, for example, to diffusion ordered NMR spectroscopy DOSY) and a small number of larger sized particles (could be either aggregates or impurities) usually overweight a larger population with smaller sizes [58].

The electrical potential between the inner particle’s surface and the bulk liquid is called Zeta potential (ζ). It is a parameter that indicates the colloidal system’s potential stability by representing the charge of a particle. Having a large positive or negative zeta potential will cause resistance toward flocculation and agglomeration. If the particles’ zeta potential values are low, there is no strength to protect them from colliding and flocculating [59]. The AgNPs prepared in this study have a zeta potential (ζ) of −21.3 ± 7.7 mV, which indicates that the AgNPs particles will have high colloidal stability [60,61,62]. The stability of AgNPs using *P. argentea* is expected to affect the distribution of NPs homogenously within the polymeric membranes.

**Figure 3:**
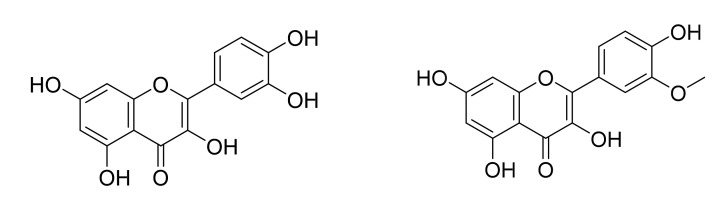
Quercetin and Isorhamnetin structures.

The chemical constituents of Jordanian *P. argentea* have been studied extensively. The plant is rich with phenolic, flavonoids, and oleanane saponins [63,64]. In addition, quercetin and isorhamnetin are the two most well-known flavonoids available in *P. argentea* [65], Figure 3.

One suggested mechanism for reducing Ag^+^ ions to AgNPs is by the electron that released from the bond of OH, which is broken from the flavonoid enol and phenolic compounds [66]. Another study suggested that the key for production of AgNPs are the flavonoids and phenolic compounds in the plant extract [67]. Flavonoids act as reducing agents for the AgNPs biosynthesis, as illustrated in the below FTIR results. Furthermore, saponins abound in *P. argentea* may act as stabilizing agents by preventing AgNPs from aggregation [68]. In this work, it was noticed that within the first three months after preparation, the synthesized AgNPs particle size did not change, indicating enhanced stability of AgNPs, which can be attributed to both the negative charge and presence of saponins.

#### 3.1.2. Transmission Electron Microscope (TEM)

**Figure 4:**
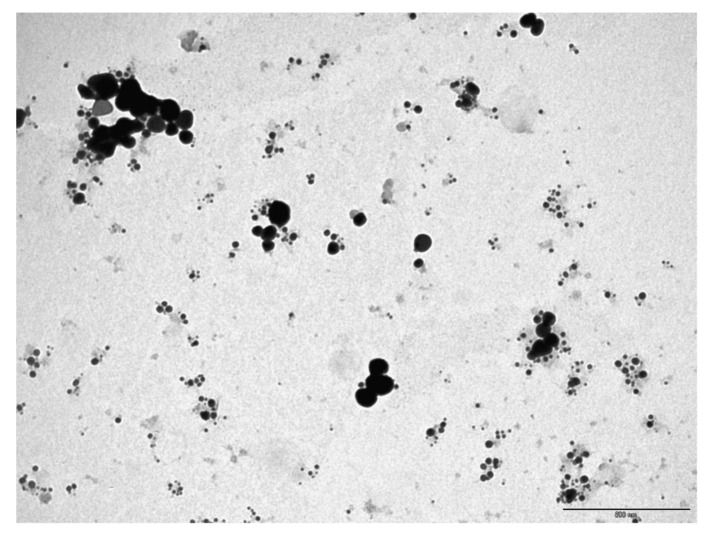
The TEM image of AgNPs. (Scale bar 500 nm.)

**Figure 5:**
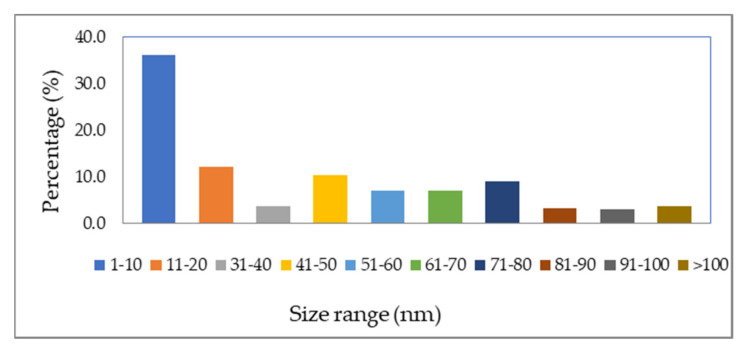
The AgNPs diameters distribution using TEM image.

The AgNPs’ shape is spherical, as shown in TEM images (Figure 4), with sizes ranging from 10 nm to more than 200 nm. These results agree with DLS data. Figure 5 shows the NPs’ diameter distribution, which was determined using the TEM image by J Microvision software, and it is evident that the particles with diameter less than 10 nm have the highest population. Similar results of the average particle size of 10 nm using TEM for AgNPs were produced by the photochemical method (ionic liquid microemulsions) [69]. Some researchers suggest that AgNPs with spherical shapes have a high surface-to-volume ratio, which increase its association with bacteria cell walls, giving better antibacterial activity [70].

#### 3.1.3. Fourier Transform Infrared Spectroscopy (FTIR)

**Figure 6:**
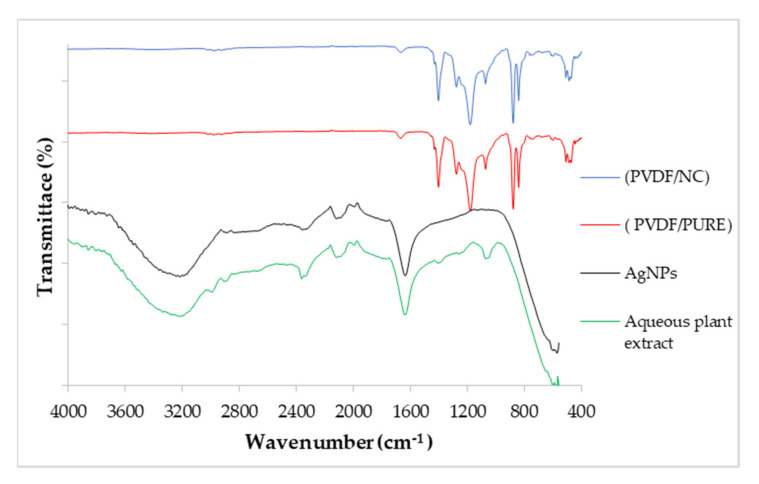
FTIR for *P. argentea* aqueous extract, AgNPs, PVDF/PURE membrane, and PVDF/NC membrane.

FTIR spectra for *P. argentea* aqueous extract, AgNPs, PVDF/PURE membrane, and PVDF/NC membrane are shown in Figure 6. The spectrum of *P. argentea* aqueous extract demonstrates peaks at (3200–3150 cm^−1^) for O-H stretching, at (2940–2915 cm^−1^) for C-H stretching in carbonyl compounds, at (2880–2830 cm^−1^) for C-H stretching in ether, at (1600–1550 cm^−1^) for C=O stretching in carboxylic compounds, at (1360–1330 cm^−1^) for C-CO-C stretching, and at (1075–1000 cm^−1^) for C-O stretching [69]. These IR bands are consistent with functional groups present in the main constituents of *P. argentea*. The IR spectrum of *P. argentea* after Ag^+^ ions conversion to AgNP is expected to retain the main characteristics bands of the plant extract (Figure 6). The peak shifts observed for the bands at 3200–3150 cm^−1^ and 1600–1550 cm^−1^ to lower wavenumbers can be attributed to the presence of AgNPs and the extract’s role in stabilizing of NPs. Similar results were found for AgNPs synthesized utilizing a green method by *Salvia Spinosa* extract [71].

The spectra of PVDF/PURE membrane and PVDF/NC) show the typical peaks of CH_2_ bending mode at 1403 cm^−1^; C-F stretching mode at 1180 cm^−1^; and PVDF characteristic peaks at 1275, 1174, and 880 cm^−1^ vibration bands [48,72]. The peaks at 409, 488, 614, 763, and 880 cm^−1^ are assigned to the α phase’s characteristic bands [73]. The two spectra of PVDF/PURE and PVDF/NC membranes are similar; however, during PVDF nanocomposite (PVDF/NC) membranes casting, the PVDF membrane changes color from white to uniform ivory when AgNPs are inserted, indicating that the AgNPs are effectively distributed across the membrane. Similar results of FTIR were obtained for PVDF membrane chemically binding a poly (vinyl alcohol) (PVA) layer and TiO_2_ NPs onto the membrane’s surface at the same time [48].

#### 3.1.4. X-ray Diffraction (XRD)

**Figure 7:**
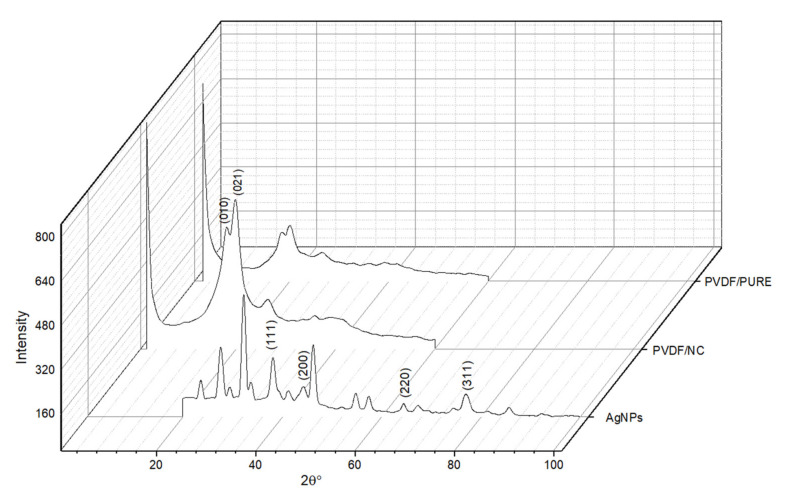
XRD for AgNPs, PVDF/PURE, and PVDF/NC membranes.

The XRD pattern for AgNPs is shown in Figure 7, with four principle peaks at 2ϴ values of 38.18, 44.16, 64.45, and 77.7 degrees corresponding to the face-centered cubic structure with crystal structure planes for AgNPs (111), (200), (220), and (311) [74]. These results are similar to those obtained for synthesized AgNPs by a green method utilizing *Lysilomaacapulcensis* (*L. acapulcensis*) extract [41]. Similar XRD patterns were also found by Ravichandran et al., who used *Parkiaspeciosa* leaf aqueous extract for AgNPs synthesis [75]. The XRD was also used to investigate crystal structures of PVDF, which has five crystalline phases α, β, γ, δ, and ε. PVDF/PURE and PVDF/NC XRD diffractograms showed the same pattern, with special peaks at 18.64, 21.56, and 28.18 degrees for PVDF/PURE and 18.26, 19.86, and 26.76 degrees for PVDF/NC, indicating α (110), and α (021), respectively. The PVDF membrane phase was not affected by the addition of AgNPs; however, the spectra show that PVDF/NC is more intense than PVDF/PURE, which is in agreement with data obtained by Kim et al. 2002 [76]. The intensity of XRD pattern for PVDF membranes was found to increase as the percentage of AgNPs increases [77], which may be attributed to enhanced crystallinity.

In another study utilizing PVDF blended with its grafted polymers (PVDF-g-poly(3-trimethoxysilylpropylmethacrylate) and PVDF-g-polyvinyl acetate), membranes with varied percentages of AgNPs (0, 0.5, 1, 2, and 4%) saw the intensity of the XRD pattern on PVDF membranes increase as the percent of AgNPs increased [77].

### 3.2. Antibacterial Activity

#### 3.2.1. Microdilution Assay

**Figure 8:**
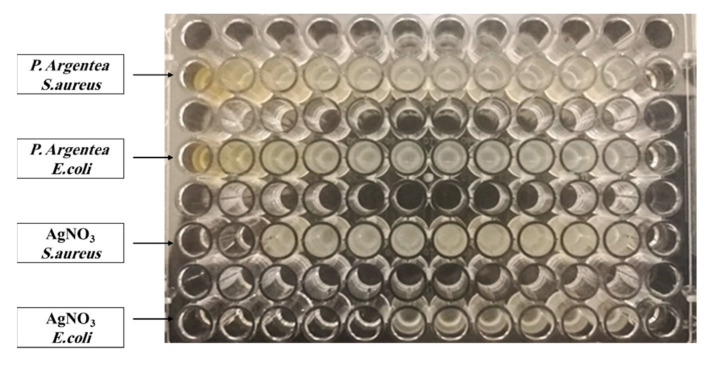
Microdilution assay against *E. coli* and *S. aureus* for the aqueous extract of *P. argentea* and AgNO_3_.

**Figure 9:**
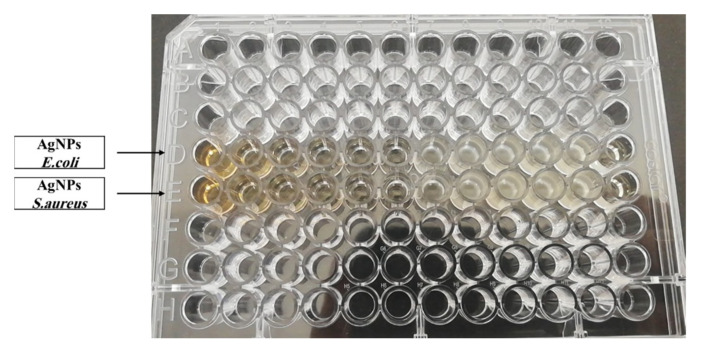
Microdilution assay against *E. coli* and *S. aureus* for of AgNPs.

MIC and MBC tests (Figure 8 and Figure 9) have been performed against *S. aureus* and *E. coli*, which are pathogenic bacteria for the aqueous extract of *P. argentea*, AgNO_3_, and AgNPs:

According to the results in the Table 3, AgNPs have a high antibacterial activity *P. argentea*, whereas there is no antibacterial activity.

The main difference of a Gram (+ve) and (−ve) bacteria is the cell walls. Gram (+ve) bacteria have one thick, peptidoglycan layer with a thickness of 20 to 80 nm and one cytoplasmic membrane; however, Gram (−ve) bacteria have two cell membranes and one thin peptidoglycan layer with a thickness of 5–10 nm [78], so the antibacterial activity is expected to differ from one to another.

For *S. aureus*, the MIC and MBC results showed better effect of AgNPs compared to that of AgNO_3_. On the other hand, for *E. coli* MIC results showed better effect of AgNPs than AgNO_3_, but MBC results were found better for AgNO_3_.

As the particles size decreases, the antibacterial activity increases, resulting from the increase in surface area. This makes it possible for more interactions with the surrounding environment [79], with the ability to enter cells, generate reactive oxygen species (ROS) and free radicals, and act as modulators in microorganism signal transduction pathways [80]. Furthermore, studies have revealed that AgNPs with spherical shape have a high surface-to-volume ratio that allows them to connect with bacteria’s cell walls, resulting in an increase in antibacterial effect [70].

Table 4 illustrates previous synthesis methods of AgNPs, their average sizes and antimicrobial activity compared to our method and results. Our results suggest that AgNPs produced by *P. argentea* aqueous extract have good antibacterial properties compared to AgNPs prepared using other plant extracts or methods.

#### 3.2.2. Antibacterial Test for PVDF Membrane

The antibiofouling activity of PVDF/NC membrane against Gram-negative (*E. coli*) and Gram-positive (*S. aureus*) bacteria was significant, according to the results shown in Table 5.

The results shown in Table 5 show that the addition of AgNPs to the membrane matrix give a very good antibacterial activity. The sterilization ratio indicates that the (PVDF-NC) membrane is antibacterial and antibiofouling membrane.

According to the literature, many studies prove that blending PVDF membrane or other polymer membrane with AgNPs decreased membrane biofouling. In these studies, antibacterial tests were evaluated using various methods such as diffusion inhibition zone method against Gram-positive and Gram-negative bacteria [7,43,77].

#### 3.2.3. SEM

**Figure 10:**
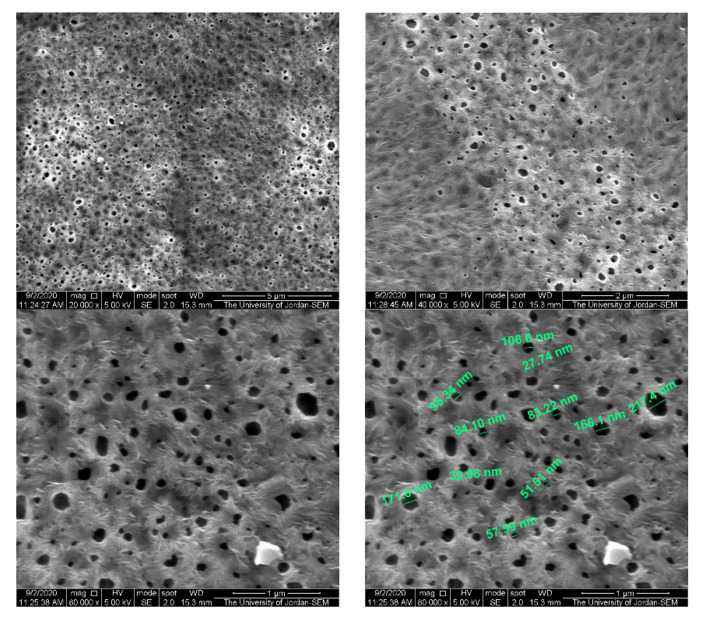
The PVDF/PURE membrane surface morphology using SEM.

**Figure 11:**
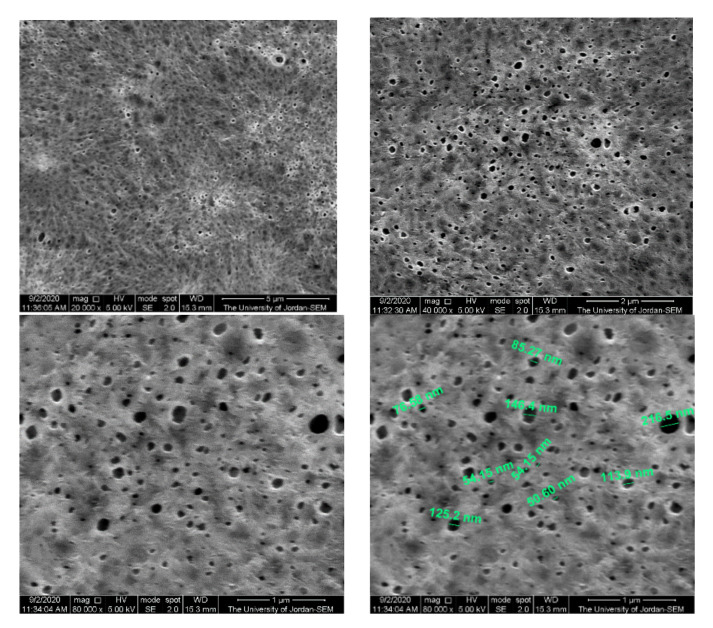
The PVDF/NC membrane surface morphology using SEM.

Figure 10 and Figure 11 show membrane surface morphology for pure PVDF in terms of pore distribution and pore size. When comparing the top surfaces of the PVDF/PURE and the PVDF/NC, as seen in the figures below, there is not much of a difference.

According to the literature, membranes with various percentages of AgNPs (0, 0.5, 1, 2, and 4%) that were produced using PVDF combined with its grafted polymers (PVDF-g-poly(3-trimethoxysilylpropylmethacrylate) and PVDF-g-polyvinyl acetate), the SEM results for cross section images showed that the structure of the PVDF membrane was not altered much when the AgNP concentration was less than 2% [77].

In another study, PVDF membrane loaded with AgNPs was prepared and showed that the size of the surface pore had slightly changed, probably due to the creation of a thin layer on the membrane, and the AgNPs were evenly distributed across the membrane’s surface, according to the SEM observations [43].

#### 3.2.4. The Hydrophilicity of Membranes

**Figure 12:**
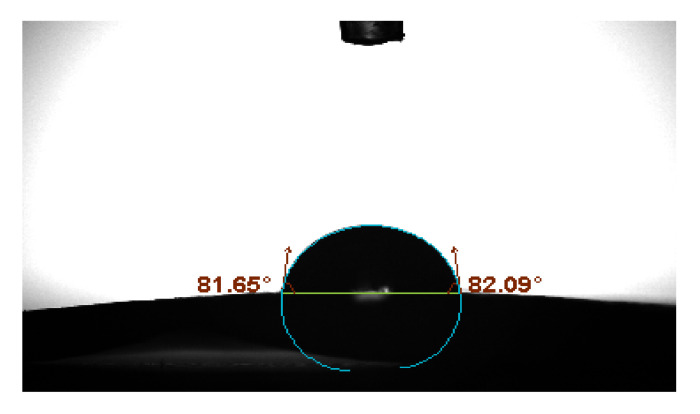
PVDF/PURE membrane contact angle.

**Figure 13:**
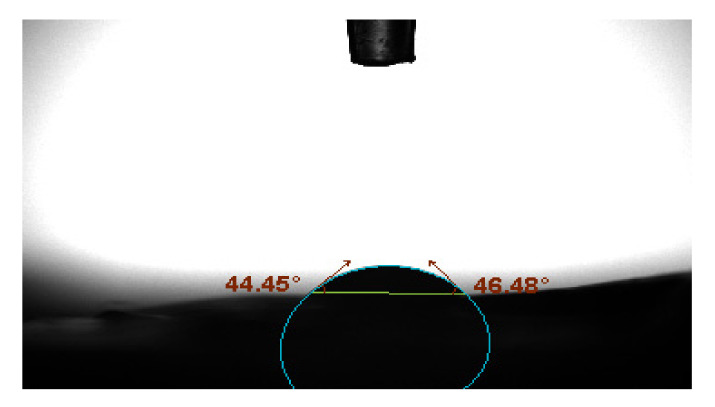
PVDF/NC membrane contact angle.

Membrane biocompatibility and antifouling performance are influenced by the surface hydrophilic/hydrophobic characteristics [82,83]. Figure 12 and Figure 13 represent the contact angles of PVDF/PURE and PVDF/NC membranes.

According to the results, the enhanced membrane’s contact angle reduced with treatment by AgNPs, indicating that the PVDF/NC membranes have more hydrophilic surface compared to PVDF/PURE membranes. AgNP aggregation on the membrane surface at relatively high concentrations is likely to be the cause for changing the membrane hydrophilicity [84].

Similar results were found for the effect of AgNPs on enhancing PVDF membrane’s hydrophilicity [83].

#### 3.2.5. PVDF Membranes Permeation Experiments

**Figure 14:**
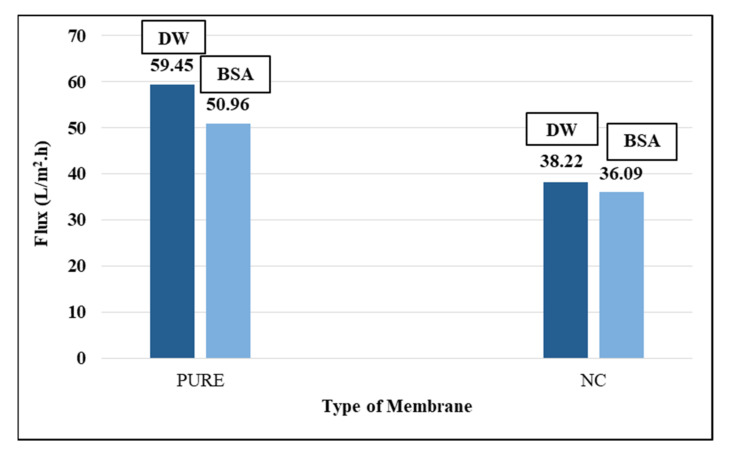
The membrane flux for PVDF and PVDF (NC) membranes.

DW and BSA aqueous solution were used in the filtration experiments, as shown in Figure 14. Dead-end stirred cell was used to measure the flux for the two membranes PVDF/PURE and PVDF/NC.

Although SEM results showed little variation in the size of the pores on the surface of the PVDF/PURE membrane compared to the PVDF/NC membrane, the rate of flux for PVDF/NC decreased compared to PVDF/PURE. It is possible that the impurities in the plant extract blocked up the membrane pores. Another point that may cause the decrease in flux is the effect of AgNPs, which influenced the free movement of polymer molecules, causing blocking of polymer microcrystal growth and conglomeration and increasing the density of the crystal nucleus; consequently, it is expected that the overall amount of crystals increased while their dimension decreased, and the voids between them become smaller over time, causing the flux to decrease, meaning that the DW flux is influenced not only by the porosity of membrane but also by the crystallinity of membrane [84].

The rejection of BSA results was 98.7% for PVDF/PURE and 99.4% for PVDF/NC.

Li et al. 2013 used AgNPs to enhance PVDF membrane’s surface hydrophilicity and antifouling performance. To investigate the impact of AgNPs on PVDF membrane, AgNPs were successfully grafted on the surface of the membrane in the presence of poly (acrylic acid) (PAA). The results indicated that the composite membrane permeability made of Ag/PVDF-g-PAA was less effective than PURE/PVDF membrane and did not improve when it comes to hydrophilicity [83].

#### 3.2.6. AgNPs Release from Membranes

The release of silver nanoparticles into water from both membranes was tested by ICP/AES. AgNPs were not detected in water after soaking PVDF/NC membrane in DW for two months or after permeability tests.

#### 3.2.7. Porosity

The average % porosity for three samples of PVDF/PURE and three samples of PVDF/NC was 82.4% and 85.3%, respectively. The results indicated a slight increase in PVDF membrane % porosity after membrane modification with AgNPs.

## 4. Conclusions

AgNPs were successfully synthesized utilizing the aqueous extract of *P. argentea*. Flavonoid compounds in the *P. argentea* act as reducing agents, and the saponins act as stabilizing agents. It was also found that AgNPs with diameter less than 10 nm demonstrated strong antibacterial effect against *S. aureus* bacteria (G + ve) and *E. coli* (G − ve). The blended AgNPs with PVDF matrix membrane (PVDF-NC), which is used as pretreatment in desalination and wastewater treatment, also exhibited antibacterial property against both bacterial strains with a sterilizing ratio of 99.9% that enhanced the membrane antibiofouling behavior. Flux experiments revealed small enhancements in BSA rejection for PVDF/NC compared to PVDF/PURE. However, the rate of flux for PVDF/NC decreased when compared to PVDF/PURE due to plant extract impurities.

## Figures and Tables

**Table 1:** The volume of added aqueous *P. argentea* extract and the percentage of conversion of Ag^+^ to AgNPs.

The Aqueous *P. argentea* Extract Volume (mL)	The Conversion Percentage of Ag^+^ to AgNPs
6.0	57.4%
8.0	83.2%
10.0	67.8%
12.0	69.0%

**Table 2:** Effect of magnetic stirrer rotation velocity on the Ag^+^ to AgNP conversion percentage (keeping the volume aqueous *P. argentea* extract fixed).

The Aqueous *P. argentea* Extract Volume (mL)	The Magnetic Stirrer Rotation Velocity (rpm)	The Conversion Percentage of Ag^+^ to AgNPs
8.0	50	78.2%
8.0	750	83.20%
8.0	1000	96.50%
8.0	1500	67.40%

**Table 3:** The antibacterial activity results for the extract of *P. argentea*, AgNO_3_, and AgNPs.

Bacteria	The Extract of *P. argentea*		AgNO_3_		AgNPs	
	MIC (μL/mL)	MBC (μL/mL)	MIC (μL/mL)	MBC (μL/mL)	MIC (μL/mL)	MBC (μL/mL)
*S. aureus* ATCC no. 25193	N.D	N.D	80.9	80.9	4.9	4.9
*E. coli* ATCC no. 8739	N.D	N.D	5.05	5.05	4.9	19.9

**Table 4:** Synthesis of AgNPs using various methods, as well as their antimicrobial activity, from previous studies compared to ours.

Method of Synthesis AgNPs	Average Size (nm) of AgNPs	Antimicrobial Activity	Reference
Photoreduction	10 nm by TEM.	By the solution of 1.56 μg/mL, AgNPs were killed over 99% of (*E. coli*) BL21.	[69]
A commercial green tea extract	34.68 ± 4.95 nm by DLS.	Various concentrations of AgNPs (2-fold dilution: 1000–0.1 µg/mL) were used in each well. *S. aureus*; ATCC 29213→ MIC = 250 µg/mL. MBC = 250 µg/mL. *E. coli*; ATCC 25922→ AgNPs: MIC = 15 µg/mL. MBC = 15 µg/mL.	[53]
Microemulsion method	The TEM average size of AgNPs was (8–40) nm.	*E. coli*; MTCC 443→ 40 nm AgNPs MIC = 100 µg/mL. 30 nm AgNPs MIC = 75 µg/mL. 8 nm AgNPs MIC = 50 µg/mL.	[81]
Our Work Green synthesis of AgNPs by the aqueous extract of *P. argentea*	The average sizes were of DLS 71.1 nm. The results of TEM showed that the highest percentage of diameter is less than 10 nm.	The solution of AgNPs (312.5 µL/mL) S. aureus; ATCC 25193 → MIC = 4.9 µg/mL. MBC = 4.9 µg/mL. E. coli; ATCC 8739 → MIC = 4.9 µg/mL. MBC = 19.9 µL/mL.	

**Table 5:** Antibacterial activity of PVDF membranes.

Membrane	Type	(*E. coli*) Bacteria (ATCC no. 8739)	(*S. aureus*) Bacteria (ATCC no. 25913)
PVDF	Control (PVDF-PURE)	48.9 × 10^6^ Bacteria/cm^2^	1.7 × 10^6^ Bacteria/cm^2^
PVDF	Treatment with AgNPs (PVDF-NC)	10 Bacteria/cm^2^	10 Bacteria/cm^2^
Sterilization ratio		99.9%	99.9%

## Data Availability

We did not report any data from this study.
